# *Hydatigera parva* population genetics in Iberian rodents provides insights into its introduction from Africa

**DOI:** 10.1017/S0031182025000058

**Published:** 2025-02

**Authors:** Milan Miljević, Marija Rajičić, Javier Marco, Jelena Blagojević, Ruth Rodríguez-Pastor, Branka Bajić, Javier Millán

**Affiliations:** 1 Department of Genetic Research, Institute for Biological Research ‘Siniša Stanković’—National Institute of the Republic of Serbia, University of Belgrade, Belgrade, Serbia; 2 Departamento de Patología Animal, Facultad de Veterinaria, Universidad de Zaragoza, Zaragoza, Spain; 3 Instituto Agroalimentario de Aragón-IA2 (Universidad de Zaragoza-CITA), Zaragoza, Spain; 4 Fundación ARAID, Zaragoza, Spain; 5 Facultad de Ciencias de la Vida, Universidad Andres Bello, Santiago, Chile

**Keywords:** *Apodemus*, Europe, *Hydatigera*, Iberia, rodents

## Abstract

This study investigated the prevalence and genetic diversity of *Hydatigera parva* in 341 native rodents in a riparian habitat in the Mediterranean part of Spain. Polycephalic larvae were found in 32% of wood mice (*Apodemus sylvaticus; n* = 84) and 0.4% of Algerian mice (*Mus spretus; n* = 257) examined, with a significantly higher prevalence in the former. No significant differences in infection prevalence in wood mouse were found between sex and age groups, habitats (agricultural vs natural) or seasons. Genetic analysis of 25 cysts using *cox1* sequences revealed low nucleotide (0.00110) and haplotype diversity (0.380), suggesting limited genetic variation. Phylogenetic analysis showed that the studied *H. parva* isolates were genetically distinct from other species within the genus *Hydatigera*. The results indicate a lower genetic diversity in European than in African populations, supporting the hypothesis that *H. parva* was introduced to Europe from Africa, possibly with its final host, the common genet (*Genetta genetta*), an abundant predator in the study area. This study is the first population genetic study of *H. parva* in the Iberian Peninsula. It provides insights into the population structure of the parasite and its interaction with rodent hosts, and thereby constitutes an example of the potential identification of an introduction route of a parasite with its definitive host. It also confirms the importance of the wood mouse as an intermediate host for the maintenance of the parasite’s life cycle in Europe and forms the basis for further studies on the distribution and genetic diversity of *H. parva*.

## Graphical abstract

**Figure:**
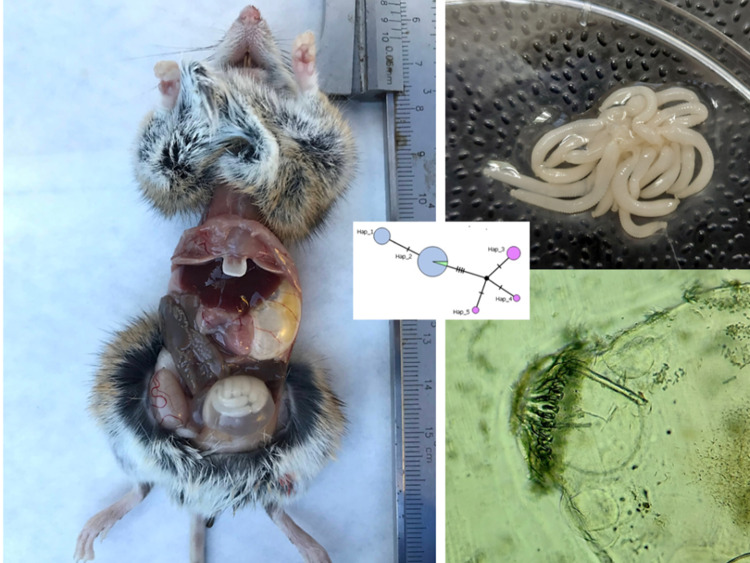

## Introduction

Cestodes of the family Taeniidae Ludwig, 1886, are parasites of terrestrial mammals that typically occur as adult tapeworms in predatory definitive hosts, with their larval stages developing in the prey. These parasitic organisms go through a life cycle that includes 2 hosts: carnivorous or omnivorous definitive hosts, which harbour the adult tapeworms in their small intestine, and herbivorous or omnivorous intermediate hosts, in which the larval stages develop in muscles, visceral organs, body cavities or the central nervous system, depending on the cestode species. The family Taeniidae, belonging to the order Cyclophyllidae, comprises 4 genera: *Taenia* Linnaeus, 1758; *Echinococcus* Rudolphi, 1801; *Hydatigera* Lamarck, 1816 and *Versteria* (Nakao et al., 2013). *Hydatigera*, a genus recently revived by Nakao et al. (2013), comprises 4 recognized species: *Hydatigera taeniaeformis* s.s. (Batsch, 1786), *Hydatigera kamiyai* (Lavikainen et al., 2016), *Hydatigera parva* (Baer, 1924) and *Hydatigera krepkogorski* Schulz and Landa, 1934. *Hydatigera* tapeworms mature in the small intestine of felids and viverrids, while their larval stages, known as metacestodes or strobilocerci, develop in the tissues and body cavities of rodents (Nakao et al., 2013; Lavikainen et al., 2016). *Hydatigera taeniaeformis* s.s. and *H. kamiyai* have been the focus of extensive research in recent years, leading to increased molecular studies and the discovery of cryptic species (Lavikainen et al., 2016). In contrast, much less attention has been paid to *H. parva* and *H. krepkogorski*, and very little is known about their genetic data, especially in the case of *H. parva*.

*H. parva* is thought to have originated in Africa and to have been introduced to the Iberian Peninsula (South-Western Europe) together with its final host, the common genet (*Genetta genetta* L. 1758; Carnivora: Viverridae). In this new environment, it continued using the same intermediate host, the wood mouse (*Apodemus sylvaticus* L. 1758), previously used in North Africa (Alvarez et al., 1990). The introduction of the common genet from North Africa to Europe probably occurred through several events facilitated by different civilizations, although the exact timing and pathways are still under debate (Gaubert et al., 2015; Delibes et al., 2017). Currently, stable populations of common genet are established in the Iberian Peninsula, Southwestern France and the Balearic Islands (Calzada, 2007; Jennings and Veron, 2009; Delibes et al., 2017). The life cycle of *H. parva* in Europe appears to revolve primarily around the genet and the wood mouse, as this parasite is detected mostly in these hosts (Alvarez et al., 1990; Fuentes et al., 2000, 2003, 2004; Torres et al., 2003; Eira et al., 2006; Millán and Casanova, 2007; Ribas et al., 2009), with occasional findings in the house mouse (*Mus musculus*) Schwarz and Schwarz, 1943 (Alvarez et al., 1987). Most of the papers referred to above have focused primarily on a wide range of parasite species and have only tangentially mentioned the presence of *H. parva*. Additionally, much of this research is over 15 years old, and genetic studies of *H. parva* in the Iberian Peninsula are limited to a single isolate (TpaSp) available in GenBank (Lavikainen et al., 2008; Nakao et al., 2013). One of the few recent studies focusing on the epidemiological and molecular findings of *H. parva* was conducted in Senegal representing the first application of genetic tools to characterize *H. parva* in autochthonous rodents on the African continent (Catalano et al., 2019).

In general, information on the occurrence of *H. parva* in the Iberian Peninsula and elsewhere in Europe is limited and sporadic, with a notable lack of molecular-genetic studies. In this context, the aims of the present study were: (i) to determine the presence of *H. parva* in populations of autochthonous rodents in Spain; (ii) to investigate distribution patterns, prevalence rates and the influence of biotic factors on infection prevalence, in order to determine whether there is any correlation between transmission patterns of the parasite and specific habitats and host characteristics; and (iii) to perform a comprehensive analysis based on mtDNA genes (*cox1*, 12S DNA), which will contribute to a better understanding of its phylogenetic relationships and provide molecular data for further studies.

## Material and methods

### Field methods

Fieldwork was carried out in 2022 in riparian habitats along the Ebro River in La Cartuja Baja, Zaragoza province, Autonomous Region of Aragón (Northwest Spain; 41°36′16″N 0°49′21″W; **Figure S1**). Two types of habitats were selected: non-protected natural riparian habitats and agricultural fields close to the natural areas. These forests are characterized by ash trees (*Fraxinus* sp.), black poplars (*Populus* sp.), willow trees (*Salix* sp.), tamarisk shrubs (*Tamarix gallica*) and common reed grasses (*Phragmites australis*). Crops are mostly devoted to alfalfa, wheat and barley. Rodents were trapped using Sherman traps (H.B. Sherman Traps, Inc., Tallahassee, Florida). A mixture of wheat flour and vegetable oil was used as bait and a piece of hydrophobic cotton as nesting material. Traps were set up in the evening and inspected in the morning for 4 consecutive days. Animals were transferred without handling to a plastic bag and weighed using a Pesola scale to the nearest 0.5 g and anesthetized with a combination of ketamine (Domtor©, Esteve, Barcelona, Spain) and medetomidine (Imalgene©, Merial, Barcelona, Spain) (Chirife and Millán, 2014). Animals were then euthanized by bleeding and necropsied in detail, sexed, aged and measured. A total of 341 individuals of 2 different species were included in the study: 257 Algerian mice (*Mus spretus* Lataste, 1883) and 84 wood mice. Parasitological specimens were preserved in 90% ethanol and transported to the laboratory of the Department of Genetic Research, Institute for Biological Research ‘Siniša Stanković’-National Institute of the Republic of Serbia, University of Belgrade, Serbia. The import of samples to Serbia was authorized by the Veterinary Directorate of the Ministry of Agriculture, Forestry and Water Management of the Republic of Serbia (permit number: 000491352 2023 14841 004 000 000 001-02).

### DNA processing: extraction, amplification and sequencing

Genomic DNA was extracted from each parasite specimen using the AccuPrep® Genomic DNA Extraction Kit (Bioneer Corporation, Daejeon, South Korea) following the manufacturer’s instructions, preceded by an overnight digestion with proteinase K and RNase. The mitochondrial cytochrome c oxidase subunit 1 (*cox1*) gene fragment (approx. 400 bp) was amplified using the primers JB3 (5′-TTT TTT GGG CAT CCT GAG GTT TAT-3′) and JB45 (5′-TAA AGAAAG AAC ATA ATG AAA ATG-3′) (Bowles et al., 1992). Additionally, a fragment of approximately 350 bp of mitochondrial (mt) 12S rDNA was amplified using the primers P60for (5′-TTA AGA TAT ATG TGG TAC AGG ATT AGA TAC CC-3′) and P375rev (5′-AAC CGA GGG TGACGG GCG GTG TGT ACC-3′) (von Nickisch-rosenegk et al., 1999). All PCRs were performed in a final reaction volume of 25 μL, which included 2.5 μL (10× PCR Dream Taq buffer), 1.25 μL dNTPs (10 mM), 1.25 μL of each primer (20 μM), 0.2 μL (1 U) Dream Taq polymerase (Thermo Fisher Scientific), genomic DNA extract (30–100 ng) and RNAse free water up to the final volume. The PCR conditions for both molecular markers were identical: an initial denaturation at 94 °C for 3 min; followed by 40 cycles of 30 sec at 94 °C, 1 min at 56 °C and 45 sec at 72 °C; and a final extension at 72 °C for 2 min. The PCR amplification products were separated by agarose gel electrophoresis, stained with Midori Green Direct (Nippon Genetics Europe) and visualized using a Bio-Rad Gel Doc 1000 (Bio-Rad Laboratories, Hercules, California, USA). Subsequently, the remaining PCR products were purified using the ExS-Pure™ Enzymatic PCR Cleanup Kit (NimaGen) and further used as templates in the sequencing reaction with the BrilliantDye® v3.1 Dye-Terminator Cycle Sequencing Kit (NimaGen), both following the manufacturer’s instructions. The sequencing reaction was followed by purification through ethanol precipitation, as suggested in the Applied Biosystems Chemistry Guide|Third Edition – DNA Sequencing by Capillary Electrophoresis (p. 76). Finally, capillary electrophoresis was performed using the SeqStudio Genetic Analyzer (Applied Biosystems – Thermo Fisher Scientific).

### Molecular, phylogenetic and genetic analysis

The DNA sequences obtained were compared with existing sequences in GenBank© using the NCBI BLAST© search tool (www.blast.ncbi.nlm.nih.gov) to confirm species identity. Subsequently, the sequences of *H. parva* (*cox1*) (344 bp) were subjected to population genetic and phylogenetic analysis with previously published sequences. Alignment and visual inspection were performed using Clustal W in MEGA software (v.11), and sequences were trimmed to a uniform length of 328 bp and 282 bp depending on the analysis type. A maximum likelihood tree was constructed with MEGA (v.11) using the HKY + G model. DnaSP 6.12.03 was used to analyse genetic diversity (including haplotype number, haplotype diversity and nucleotide diversity) and neutrality indices (Fu’s Fs and Tajima’s D) (Rozas et al., 2017). PopART 1.7 was used to create a median join network containing 25 *cox1* nucleotide sequences from our study and 7 sequences from the GenBank^©^ database (Bandelt et al., 1999). Pairwise nucleotide sequence divergences were calculated using the Kimura 2 parameter (K2P) (Kimura, 1980) model with a gamma value of 0.5 in MEGA (v.11) software.

### Statistical analysis

A binary logistic regression model was used to evaluate the association between the probability of a wood mouse being parasitized and the season, type of habitat (agricultural vs natural), and the mouse’s sex and age. A backward stepwise elimination method was used in which the least significant variable was removed after each step. Statistical analysis was performed using IBM SPSS Statistics 26 for Windows® (IBM Corporation, Route 100, Somers, New York, USA). A significance level of *P* < 0.05 was considered statistically significant.

## Results

**Figure 1.**
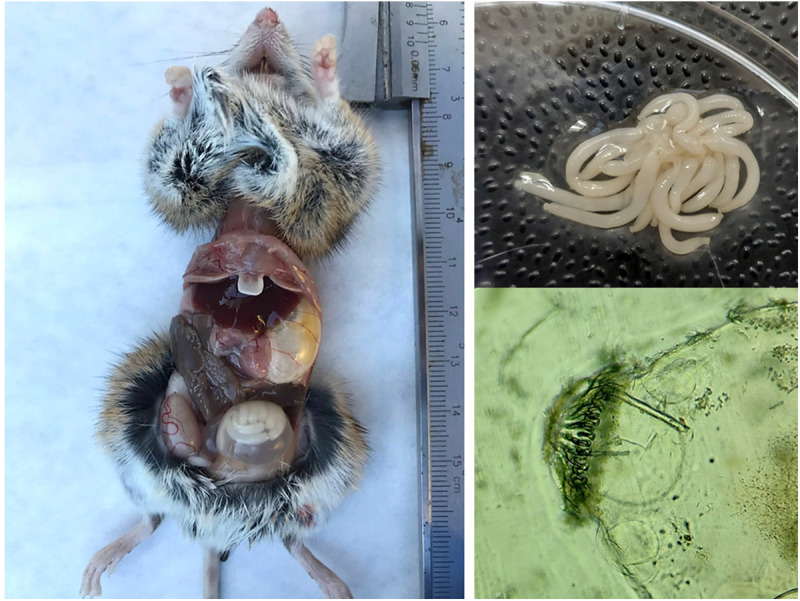
A wood mouse with an abdominal cyst (left); larvae extracted from the cyst (top right); a microscope image of the rostellum (bottom right).

Encysted polycephalic larvae of *H. parva* were found in the body cavity of 27 wood mice (32.14%) (Figure 1) and a single Algerian mouse (0.39%). Prevalence was significantly higher in wood mouse (*χ*^2^ = 84.69, *df* = 1, *p* < 0.001). All observed cysts showed the characteristic morphology of classical metacestodes, and the opening revealed the typical shape of polycephalic strobilocerci. Nine wood mice had 2 cysts, one had 3, and the remaining had 1 cyst. The cyst from the Algerian mouse had no viable larvae. The statistical analysis did not reveal any association between the presence of cysts in wood mouse and the type of habitat, the season or the animal’s sex and age.

Of the 28 genetically analysed cysts, *cox1* sequences (344 bp) were successfully amplified in 25 samples, while 3 samples were confirmed using the 12S rDNA gene, which was amplified only when *cox1* amplification was unsuccessful. All 25 *cox1* sequences and three 12S rDNA sequences of *H. parva* showed 99.31–100% identity with the sequence NC_021141 from GenBank^©^ originating from a wood mouse from Galicia, Northwestern Spain. Genetic analysis of 25 *cox1* sequences revealed a G + C content of 31.1%. A single variable site and 1 mutation were identified among the total positions analysed. Nucleotide diversity (*π*) was calculated to be 0.00110, with an average of 0.38000 nucleotide differences (*k*) observed. Haplotype analysis revealed 2 distinct haplotypes, resulting in a haplotype diversity (Hd) of 0.380. Statistical tests, including Tajima’s D (0.72124) and Fu’s Fs (1.032), showed no significant deviation from neutrality (Table 1).

**Table 1.** Genetic diversity metrics of cox1 (344 bp) sequences from this study

Indices	*Cox1* (344 bp)
No. of sequences	25
Variable (polymorphic) sites	1
Parsimony informative sites	1
Singleton variable site	1
No. of haplotypes	2
Haplotype diversity (Hd)	0.380
Nucleotide diversity (*π*)	0.00110
Nucleotide differences (*k*)*	0.38000
Tajima’s D	0.72124 (NS)
Fu’s Fs	1.032 (NS)

NS, not significant;

* Average number of pairwise nucleotide differences (*k*).

**Figure 2.**
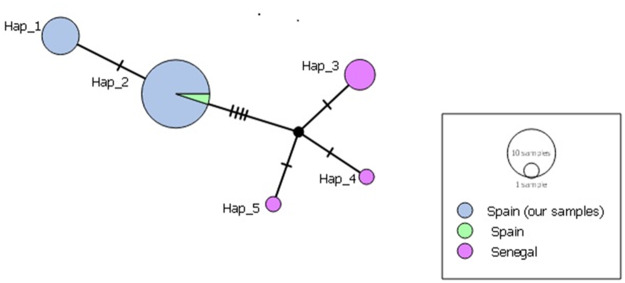
Median-joining network of *H. parva* isolates from our study compared to isolates from Africa (Senegal) and an isolate from Spain from GenBank (MH036503-MH036508; AB731760), based on *cox1* gene sequences (328 bp).

**Figure 3.**
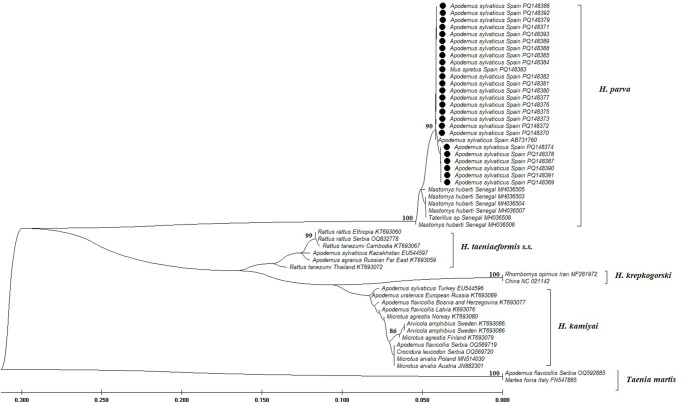
Phylogenetic tree of *H. parva* based on 282 bp *cox1* gene sequences. A maximum likelihood tree was constructed using MEGA (v.11) with the HKY + G model. Values >85% are indicated. Sequences of other *Hydatigera* species from GenBank^©^ studies are included in the tree, with *Taenia martis* used as an outgroup.

Twenty-five *cox1* sequences from our study and seven sequences available in GenBank^©^ (six from Senegal and one from Spain) were all truncated to 328 bp. According to the median-joining network analysis, these sequences revealed a total of 5 haplotypes. The single isolate from Spain in GenBank clustered with our samples, while the 6 sequences from Senegal resulted in 3 distinct haplotypes. There were 5–7 mutational steps between these 2 regional groups of haplotypes (Figure 2). Inter-population pairwise divergence analysis of *H. parva* samples from Spain revealed distance variations up to 0.30%, while regional analysis with samples from Africa (Senegal) indicated distances between 1.6% and 1.9%. At the inter-species level, our *H. parva* samples showed significant differences from congeneric samples: 17.50–18.64% from *H. kamiyai*, 16.46–20.41% from *H. taeniaeformis* s.s. and 19.19–19.74% from *H. krepkogorski* (Table 2). Phylogenetic analyses supported these results by clustering our samples with *H. parva* and identified the distances from other species within the genus *Hydatigera* (Figure 3).

**Table 2.** Pairwise genetic distances between *H. parva* and other *Hydatigera* species (sequences 282 bp)

	1	2	3	4	5	6	7	8	9	10	11	12	13	14
1 *H. parva* (*Apodemus sylvaticus*)*														
2 *H. parva* (*Apodemus sylvaticus*)*	0.36													
3 *H. kamiyai* (*Apodemus flavicollis* Serbia OQ592884)	18.04	17.50												
4 *H. kamiyai* (*Apodemus flavicollis* Serbia OQ592885)	18.04	17.50	0.00											
5 *H. kamiyai* (*Crocidura leucodon* Serbia OQ569720)	18.07	17.54	16.97	16.97										
6 *H. kamiyai* (*Arvicola amphibius* Sweden KT693086)	18.07	17.54	16.43	16.43	1.46									
7 *H. kamiyai* (*Apodemus flavicollis* Latvia K693076)	18.64	18.10	15.90	15.90	1.09	1.84								
8 *H. kamiya*i (*Apodemus flavicollis* B&H KT693077)	18.07	17.54	16.43	16.43	1.84	2.61	0.72							
9 *H. taeniaeformis* s.s. (*Rattus tanezumi* Cambodia KT693067)	19.22	18.67	15.90	15.90	10.66	10.66	11.15	12.16						
10 *H. taeniaeformis* s.s. (*Rattus tanezumi* Thailand KT693072)	16.99	16.46	15.39	15.39	10.70	10.70	10.21	10.21	4.16					
11 *H. taeniaeformis* s.s. (*Rattus rattus* Ethiopia KT693060)	19.26	18.71	15.91	15.91	10.70	10.70	11.20	12.22	0.36	4.18				
12 *H. taeniaeformis* s.s. (*Rattus norvegicus* Cambodia KT693044)	20.41	19.85	16.43	16.43	11.65	10.70	11.15	12.16	0.72	4.16	1.08			
13 *H. taeniaeformis* s.s. (*Rattus rattus* Serbia OQ832778)	19.26	18.71	15.91	15.91	10.70	10.70	11.20	12.22	0.36	4.18	0.00	1.08		
14 *H. krepkogorski* (mt genome NC021142)	19.74	19.19	15.38	15.91	9.64	9.64	10.59	10.59	12.44	11.93	12.42	11.95	12.42	
15 *H. krepkogorski* (*Rhombomys opimus* Iran MF281972)	19.74	19.19	15.38	15.91	9.64	9.64	10.59	10.59	12.44	11.93	12.42	11.95	12.42	0.00

Values represent the proportional genetic distances (substitutions per site) calculated using the Kimura 2-parameter model (K2P) with gamma correction (gamma = 0.5) (Kimura, 1980).

* The isolates from this study represent 2 different haplotypes.

## Discussion

Our results indicated a relatively high prevalence of *H. parva* in wood mice, compared to previous studies conducted in Spain and Portugal 2 decades ago, which showed lower rates ranging from 3% to 13.3% (Fuentes et al., 2000, 2003, 2004; Eira et al., 2006). This high infection rate can be attributed to the selective feeding preferences of the common genet. Despite its generalist feeding behaviour, this viverrid seems to prey predominantly on wood mice in the region (Torre Corominas et al., 2015; Belbel et al., 2022), suggesting that it may play a crucial role in maintaining the life cycle of *H. parva* in the Iberian Peninsula. In our study, *H. parva* was detected in Algerian mice in only 1 case, contrasting with the high prevalence of the parasite in wood mice, which is consistent with its recognized role as a suitable host. The hypothesis that *H. parva* has continued to utilize the wood mouse as an intermediate host since its introduction alongside the definitive host (the common genet) to the Iberian Peninsula (Alvarez et al., 1990) is supported by our findings. This specificity suggests that the parasite may not have initiated adaptation or utilization of other micromammals from the region as intermediate hosts. It is important to remark that the isolate of *H. parva* detected in the Algerian mouse in our study had no viable larvae, confirming unsuccessful cyst development in this host and suggesting incidental exposure to infective eggs.

Our examination of wood mice in Spain indicated no significant differences in *H. parva* infection prevalence between sexes, habitats (natural and agricultural) and seasons (winter, spring, summer, autumn). In contrast, a study conducted in Portugal reported significant differences in *H. parva* prevalence based on some of these factors (Eira et al., 2006). It was found that wood mouse males tend to show higher prevalence than females, and that prevalence can vary significantly among different seasons, with summer values being lower compared to other seasons. A study from Senegal (Africa) found that season significantly influenced the probability of infection, with the majority of infected *Mastomys huberti* (Wroughton, 1909) captured during spring, while prevalence of *H. parva* did not vary significantly with host gender, habitat or locality (Catalano et al., 2019). All these contrasting findings suggest that the factors influencing *H. parva* prevalence may vary significantly between different regions and environmental conditions. Additionally, the population size of the definitive host (common genet), which serves as the main reservoir for this species, likely plays an important role. It should be noted that high prevalences of *H. parva*, ranging from 50% to 100%, have been reported in the common genet in Spain (Alvarez et al., 1990; Millán and Casanova, 2007; Ribas et al., 2009).

In our study, all polycephalic larvae of *H. parva* were confirmed by amplification and sequencing using 2 mitochondrial markers (*cox1* and 12S rDNA), representing the first molecular study of this parasite from the Iberian Peninsula at the population level and one of the few genetic studies worldwide (Catalano et al., 2019). Following the comprehensive taxonomic revision of the genus *Hydatigera*, including a reclassification and the identification of cryptic species (Nakao et al., 2013; Lavikainen et al., 2016), several molecular genetic studies have subsequently focused on *H. taeniaeformis* s.s. and *H. kamiyai* in Asia and Europe (Bajer et al., 2020; Zhao et al., 2020; Alvi et al., 2021; Martini et al., 2022; Miljević et al., 2023). However, *H. parva* has remained unexplored at the genetic level. Based on *cox1* sequence analysis of the 25 *H. parva* isolates, we identified only 2 haplotypes, resulting in low haplotype diversity (Hd: 0.380). Furthermore, the only 6 sequences from Senegal (Africa), deposited in GenBank^©^, showed potentially greater diversity (3 haplotypes out of 6 sequences). This contrast indicates a potentially reduced genetic diversity after the introduction of the parasite to Europe. Therefore, our results support the hypothesis that the parasite was introduced to Europe from Africa, probably following the introduction of the genet, as it is widely known that species generally have a higher genetic diversity in their place of origin (Austerlitz et al., 1997). The pairwise divergence within our samples was up to 0.30%, while the genetic divergence with African isolates was slightly higher at 1.6–1.9%, illustrating geographic variation within the same species. This level of genetic divergence suggests a degree of genetic isolation, reflecting the effects of geographical distance between populations, likely driven by limited gene flow and temporal separation. Our *H. parva* isolates showed genetic distances to other species within the genus *Hydatigera* ranging from about 16% to 20%. This distinction confirms the precise taxonomic classification and suggests that the studied specimens are genetically distinct from other species of the genus *Hydatigera*. In addition, other authors have shown similar genetic distances within species of the genus *Hydatigera* (Mello et al., 2018; Catalano et al., 2019), further supporting our results.

In summary, the wood mouse proved to be the most suitable intermediate host species for *H. parva* in the study area, playing an important role in maintaining the parasite’s life cycle in the Iberian Peninsula. These preliminary results on the prevalence and genetic variation of *H. parva* in Spain provide valuable information for future studies on the distribution and population structure of *H. parva* in the Iberian Peninsula. In addition, this is the first population study of *H. parva*, based on mitochondrial genes in Europe and the second worldwide, representing important contribution to the understanding of genetic diversity and host suitability of this tapeworm species. Further studies are needed to better understand the genetic relationships between the populations in Africa and on the Iberian Peninsula and the evolutionary dynamics of the parasite. Our study serves as an example of the potential identification of introduction routes of a parasite with its definitive host.

## Supporting information

Miljević et al. supplementary material Miljević et al. supplementary material

## Data Availability

Nucleotide sequences of cox1 and 12S rDNA genes from the present study have been deposited in the GenBank database under the accession number PQ148369-PQ148393.

## Supplementary material

The supplementary material for this article can be found at https://doi.org/10.1017/S0031182025000058.

## References

[ref1] ‘Álvarez F, Iglesias R, Bos J, Tojo J and Sanmart’ín ML (1990) New findings on the helminth fauna of the common European genet (*Genetta genetta* L.): First record of *Toxocara genettae* Warren, 1972 (Ascarididae) in Europe. *Annales de Parasitologie Humaine Et Comparée* 65, 244–248. doi:10.1051/parasite/1990655244

[ref2] ‘Álvarez MF, Quinteiro-Alonso P, Outeda-Macias M and Sanmart’ín-Dur’án ML (1987) Larvas de cestodos en los múridos gallegos. *Revista Ibérica de Parasitología, Volumen Extraordinario*, 91–96.

[ref3] Alvi MA, Li L, Ohiolei JA, Qamar W, Saqib M, Tayyab MH, Altaf J, Ashfaq K, Hassan A, Jamal M, Wahab A, Alvi AA, Usman M, Bajwa MRK, Fu B-Q, Yan H-B and Jia W-Z (2021) *Hydatigera taeniaeformis* in urban rats (*Rattus rattus*) in Faisalabad, Pakistan. *Infection Genetics & Evolution* 92, 104873. doi:10.1016/j.meegid.2021.104873 33905888

[ref4] Austerlitz F, Jung-Muller B, Godelle B and Gouyon PH (1997) Evolution of coalescence times, genetic diversity and structure during colonization. *Theoretical Population Biology* 51, 148–164. doi:10.1006/tpbi.1997.1302

[ref5] Bajer A, Alsarraf M, Dwużnik D, Mierzejewska EJ, Kołodziej-Sobocińska M, Behnke-Borowczyk J, Banasiak Ł, Grzybek M, Tołkacz K, Kartawik N, Stańczak Ł, Opalińska P, Krokowska-Paluszak M, Górecki G, Alsarraf M and Behnke JM (2020) Rodents as intermediate hosts of cestode parasites of mammalian carnivores and birds of prey in Poland, with the first data on the life-cycle of Mesocestoides melesi. *Parasites and Vectors* 13, 95. doi:10.1186/s13071-020-3961-2 32087754 PMC7036256

[ref6] Bandelt HJ, Forster P and Rohl A (1999) Median-joining networks for inferring intraspecific phylogenies. *Molecular Biology and Evolution* 16, 37–48. doi:10.1093/oxfordjournals.molbev.a026036 10331250

[ref7] Belbel F, Boukheroufa M, Benotmane CH, Sakraoui R, Henada LRI and Sakraoui F (2022) Selection strategy of small mammalian preys by the common genet *Genetta genetta* between natural and anthropized environments in Edough forest massif (Northeastern Algeria). *Journal of Bioresource Management* 9, 35–41.

[ref8] Bowles J, Blair D and Mcmanus D (1992) Genetic variants within the genus *Echinococcus* identified by mitochondrial DNA sequencing. *Molecular and Biochemical Parasitology* 54, 165–173. doi:10.1016/0166-6851(92)90109-W 1435857

[ref9] Calzada J (2007) *Genetta genetta* (Linnaeus, 1758). In Palomo LJ, Gisbert J and Blanco JC (eds.), *Atlas Y Libro Rojo de Los Mamíferos Terrestres de España. Dirección General Para la Biodiversidad*. Madrid: SECEM-SECEMU, 330–332.

[ref10] Catalano S, Bâ K, Diouf ND, Léger E, Verocai GG and Webster JP (2019) Rodents of Senegal and their role as intermediate hosts of *Hydatigera* spp. (Cestoda: Taeniidae). *Parasitology* 146, 299–304. doi:10.1017/S0031182018001427 30152308

[ref11] Chirife AD and Millán J (2014) Field immobilization of wood mice (*Apodemus sylvaticus*) with medetomidine and ketamine and antagonism with atipamezole. *Journal of Wildlife Diseases* 50, 961–963. doi:10.7589/2013-10-261 25098298

[ref12] Delibes M, Centeno-Cuadros A, Muxart V, Delibes G, Ramos-Fernández J and Morales A (2017) New insights into the introduction of the common genet, *Genetta genetta* (L.) in Europe. *Archaeological and Anthropological Sciences* 11, 531–539. doi:10.1007/s12520-017-0548-8

[ref13] Eira C, Torres J, Vingada J and Miquel J (2006) Ecological aspects influencing the helminth community of the wood mouse *Apodemus sylvaticus* in Dunas de Mira, Portugal. *Acta Parasitologica* 51. doi:10.2478/s11686-006-0046-0

[ref14] Fuentes MV, Cerezuela AM and Galan-Puchades MT (2000) A helmithological survey of small mammals (insectivores and rodents) in the Serra Calderona mountains (Valencian Community, Spain). *Research and Reviews in Parasitology* 60, 23–36.

[ref15] Fuentes MV, Fuentes MV, Sáez S, Trelis M, Galán-Puchades MT and Esteban JG (2004) The helminth community of the wood mouse, *Apodemus sylvaticus*, in the Sierra Espuña, Murcia, Spain. *Journal of Helminthology* 78, 219–223. doi:10.1079/JOH2003226 15469624

[ref16] Fuentes MV, Sáez S, Trellis M, Cruz J, Sarmento P, Casanova JC, Torres J, Feliu C and Esteban JG (2003) Helminthofauna of small mammals (Insectivora, Rodentia) collected in tbe Serra da Malcata (Portugal). *Revista Ibérica de Parasitología* 63, 89–92.

[ref17] Gaubert P, Del Cerro I, Centeno-Cuadros A, Palomares F, Fournier P, Fonseca C, Paillat J-P and Godoy JA (2015) Tracing historical introductions in the Mediterranean Basin: The success story of the common genet (*Genetta genetta*) in Europe. *Biological Invasions* 17, 1897–1913. doi:10.1007/s10530-015-0846-y

[ref18] Jennings AP and Veron G (2009) Family Viverridae. In Wilson DE and Mittermeier RA (eds.), *Handbook of the Mammals of the World. Vol. 1. Carnivores*. Barcelona: Lynx Edicions, 174–224.

[ref19] Kimura M (1980) A simple method for estimating evolutionary rates of base substitutions through comparative studies of nucleotide sequences. *Journal of Molecular Evolution* 16, 111–120. doi:10.1007/BF01731581 7463489

[ref20] Lavikainen A, Haukisalmi V, Lehtinen MJ, Henttonen H, Oksanen A and Meri S (2008) A phylogeny of members of the family Taeniidae based on the mitochondrial *cox1* and *nad1* gene data. *Parasitology* 135, 1457–1467. doi:10.1017/S003118200800499X 18937885

[ref21] Lavikainen A, Iwaki T, Haukisalmi V, Konyaev SV, Casiraghi M, Dokuchaev NE, Galimberti A, Halajian A, Henttonen H, Ichikawa-Seki M, Itagaki T, Krivopalov AV, Meri S, Morand S, Näreaho A, Olsson GE, Ribas A, Terefe Y and Nakao M (2016) Reappraisal of *Hydatigera taeniaeformis* (Batsch, 1786) (Cestoda: Taeniidae) sensu lato with description of *Hydatigera kamiyai* n. sp. *International Journal for Parasitology* 46, 361–374. doi:10.1016/j.ijpara.2016.01.009 26956060

[ref22] Martini M, Dumendiak S, Gagliardo A, Ragazzini F, La Rosa L, Giunchi D, Thielen F, Romig T, Massolo A and Wassermann M (2022) *Echinococcus multilocularis* and other taeniid metacestodes of muskrats in Luxembourg: Prevalence, risk factors, parasite reproduction, and genetic diversity. *Pathogens* 11, 1414. doi:10.3390/pathogens11121414 36558748 PMC9781964

[ref23] Mello ÉM, Furtado LFV, Rabelo ÉML and Pinto HA (2018) DNA barcoding of metacestodes found in the *Guerlinguetus ingrami* (Rodentia: Sciuridae) reveals the occurrence of *Hydatigera taeniaeformis* sensu stricto (Cyclophyllidea: Taeniidae) in the Americas. *Parasitology International* 67, 115–118. doi:10.1016/j.parint.2017.10.005 29055694

[ref24] Miljević M, Rajičić M, Umhang G, Bajić B, Bjelić Čabrilo O, Budinski I and Blagojević J (2023) Cryptic species *Hydatigera kamiyai* and other taeniid metacestodes in the populations of small mammals in Serbia. *Parasites and Vectors* 16, 250. doi:10.1186/s13071-023-05879-x 37491284 PMC10369706

[ref25] Millán J and Casanova JC (2007) Helminth parasites of the endangered Iberian lynx (*Lynx pardinus*) and sympatric carnivores. *Journal of Helminthology* 81, 377–380. doi:10.1017/S0022149X07869203 18021466

[ref26] Nakao M, Lavikainen A, Iwaki T, Haukisalmi V, Konyaev S, Oku Y, Okamoto M and Ito A (2013) Molecular phylogeny of the genus *Taenia* (Cestoda: Taeniidae): Proposals for the resurrection of *Hydatigera* Lamarck, 1816 and the creation of a new genus *Versteria*. *International Journal for Parasitology* 43, 427–437. doi:10.1016/j.ijpara.2012.11.014 23428901

[ref27] Ribas A, Feliu C and Casanova J (2009) Distribution of the cestode *Taenia parva* (Taeniidae) along the digestive tract of the common genet (*Genetta genetta*). *Helminthologia* 46, 35–38. doi:10.2478/s11687-009-0007-x

[ref28] Rozas J, Ferrer-Mata A, Sánchez-delbarrio JC, Guirao-Rico S, Librado P, Ramos-Onsins SE and Sánchez-Gracia A (2017) DnaSP 6: DNA sequence polymorphism analysis of large data sets. *Molecular Biology and Evolution* 34, 3299–3302. doi:10.1093/molbev/msx248 29029172

[ref29] Torre Corominas I, Arrizabalaga A and Ribas A (2015) The diet of the genet (*Genetta genetta* Linnaeus, 1758) as a source of information on local small mammal communities. *Galemys, Spanish Journal of Mammalogy* 27, 71–75. doi:10.7325/Galemys.2015.N4

[ref30] Torres J, Trelis M, Espert A, Ribas A, Toledo R, Casanova J, Roman J, Arrizabalga A, Esteban JG and Feliu C (2003) Helminth fauna of small mammals (insectivores and rodents) in Doñana (southeastern Iberian Peninsula). *Revista Ibérica de Parasitología* 63, 23–29.

[ref31] von Nickisch-rosenegk M, Silva-Gonzalez R and Lucius R (1999) Modification of universal 12S rDNA primers for specific amplification of contaminated *Taenia* spp. (Cestoda) gDNA enabling phylogenetic studies. *Parasitology Research* 85, 819–825. doi:10.1007/s004360050638 10494807

[ref32] Zhao F, Zhou Y, Wu Y, Zhou K, Liu A, Yang F and Zhang W (2020) Prevalence and genetic characterization of two mitochondrial gene sequences of *Strobilocercus fasciolaris* in the livers of brown rats (*Rattus norvegicus*) in Heilongjiang Province in Northeastern China. *Frontiers in Cellular & Infection Microbiology* 10, 588107. doi:10.3389/fcimb.2020.588107 33324575 PMC7723829

