# Supplementary figures and images for: Hydatigera parva population genetics in Iberian rodents provides insights into its introduction from Africa
Source: Parasitology. 2025 Jan 22;152(2):149–55. doi: 10.1017/S0031182025000058 (PMC12089443; doi:10.1017/S0031182025000058)

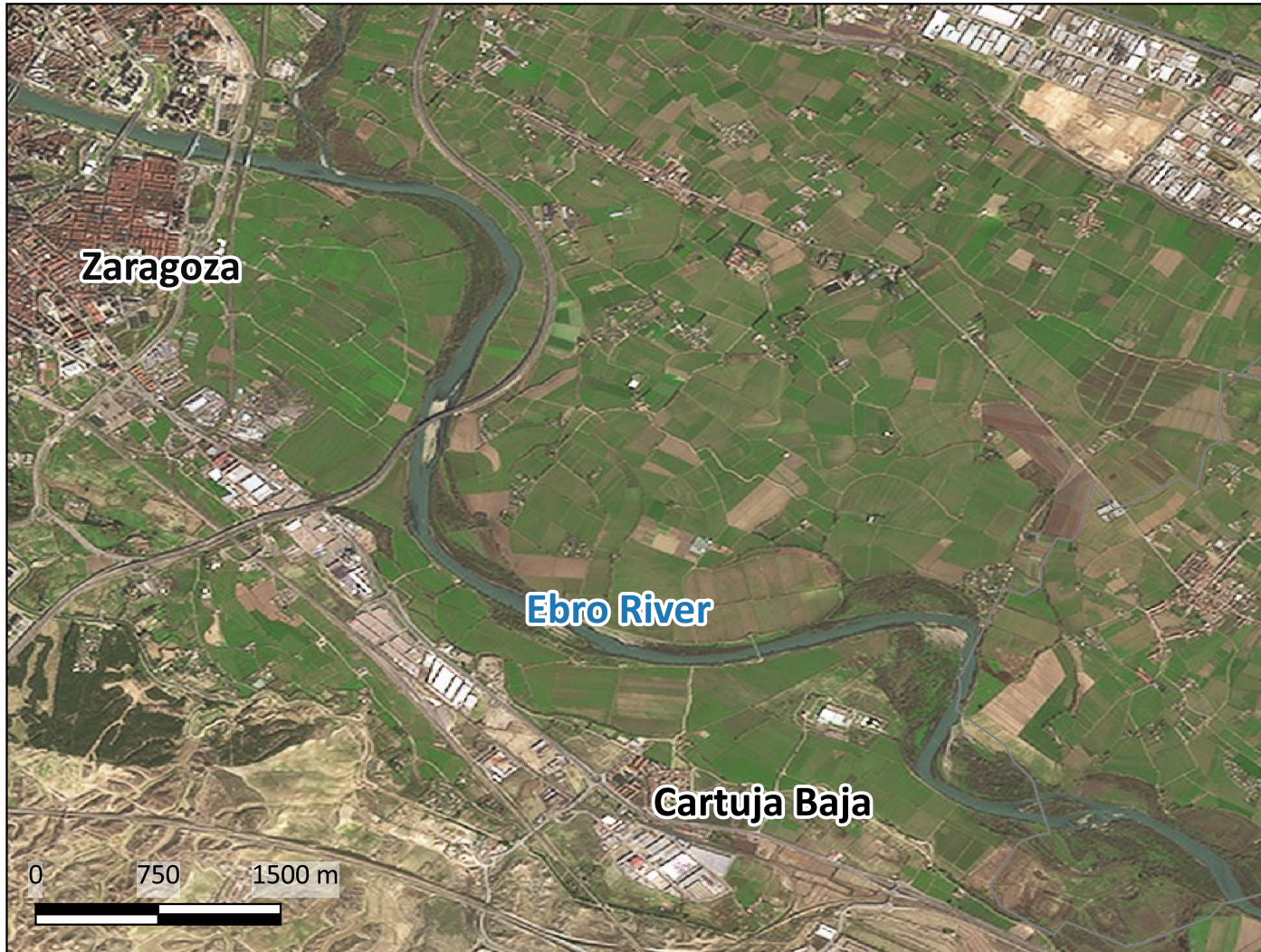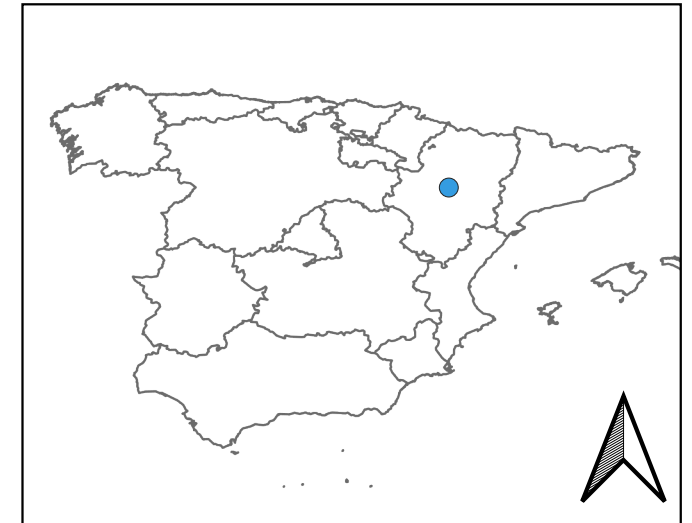

Supplement: Miljević et al. supplementary material [file S0031182025000058sup001.pdf]
